# Risk factors for measles among adults in Tianjin, China: Who should be controls in a case-control study?

**DOI:** 10.1371/journal.pone.0185465

**Published:** 2017-09-26

**Authors:** Abram L. Wagner, Matthew L. Boulton, Brenda W. Gillespie, Ying Zhang, Yaxing Ding, Bradley F. Carlson, Xiaoyan Luo, JoLynn P. Montgomery, Xiexiu Wang

**Affiliations:** 1 Department of Epidemiology, School of Public Health, University of Michigan, Ann Arbor, Michigan, United States of America; 2 Department of Internal Medicine, University of Michigan Medical School, Ann Arbor, Michigan, United States of America; 3 Department of Biostatistics, School of Public Health, University of Michigan, Ann Arbor, Michigan, United States of America; 4 Institute of Human Communicable Diseases Control and Prevention, Tianjin Centers for Disease Control and Prevention, Tianjin, China; TNO, NETHERLANDS

## Abstract

**Background:**

Control groups in previous case-control studies of vaccine-preventable diseases have included people immune to disease. This study examines risk factors for measles acquisition among adults 20 to 49 years of age in Tianjin, China, and compares findings using measles IgG antibody-negative controls to all controls, both IgG-negative and IgG-positive.

**Methods:**

Measles cases were sampled from a disease registry, and controls were enrolled from community registries in Tianjin, China, 2011–2015. Through a best subsets selection procedure, we compared which variables were selected at different model sizes when using IgG-negative controls or all controls. We entered risk factors for measles in two separate logistic regression models: one with measles IgG-negative controls and the other with all controls.

**Results:**

The study included 384 measles cases and 1,596 community controls (194 IgG-negative). Visiting a hospital was an important risk factor. For specialty hospitals, the odds ratio (OR) was 4.53 (95% confidence interval (CI): 1.28, 16.03) using IgG-negative controls, and OR = 5.27 (95% CI: 2.73, 10.18) using all controls. Variables, such as age or length of time in Tianjin, were differentially selected depending on the control group. Individuals living in Tianjin ≤3 years had 2.87 (95% CI: 1.46, 5.66) times greater odds of measles case status compared to all controls, but this relationship was not apparent for IgG-negative controls.

**Conclusions:**

We recommend that case-control studies examining risk factors for infectious diseases, particularly in the context of transmission dynamics, consider antibody-negative controls as the gold standard.

## Introduction

Successful efforts in global measles control have dramatically decreased one of the leading causes of communicable mortality worldwide to less than 150,000 deaths in 2013 [1,2]. As the world’s most populous country, China faces a uniquely high burden of measles despite substantial governmental investment in control efforts. A national goal to eliminate measles by 2012 was not met and there were increases in the number of measles cases in 2013 and 2014 [3]. As China approaches its measles end game, progressively more cases are occurring in adults whose vaccination status is unknown [3].

Vaccination is the key preventive intervention for measles control. Nationwide, China has had a two-dose measles-containing vaccine (MCV) schedule since 1986 [4], and some provincial-level units, like Tianjin, have introduced a third dose within the past decade. Vaccination records prior to 2000 are scarce, though, and most adults have an unknown vaccination status [5], diminishing the usefulness of vaccination history as a proxy for measles immunity in Chinese adults.

Reported risk factors for measles among adults in China include exposure to hospitals [6–8], and poverty [7,9]. Migration to cities was shown in some studies [7,9] but not others [10] to increase the odds of acquiring measles. Travel between cities was positively related to measles acquisition in one study [9] but not another [8]. The generalizability of these results is uncertain given their small sample sizes, short study durations, and nonspecific definitions (e.g. “hospital” vs specific types of hospitals) [6,8–10].

Additionally, there is the question to who is an appropriate control in these case-control studies. The conventional definition specifies that “controls should be selected from the same population—the source population—that gives rise to the study cases” [11]. Thus, individuals who are measles immune, either through full vaccination or prior disease, should be ineligible to be controls since they are not at risk for disease acquisition. Conversely, those who are measles IgG negative and not immune represent the purest group of controls, although they are more logistically difficult to enroll into a study. There are potentially many (unmeasured) socioeconomic status and behavioral risk factors associated with those who are measles immune and those who are not.

The purpose of this study was two-fold: to examine risk factors for acquisition of measles among adults in Tianjin, China, and to compare these findings using controls who were measles IgG antibody negative versus using all controls (both IgG-negative and IgG-positive).

## Materials and methods

Tianjin is one of 4 province-level municipalities in China. Its 15.2 million residents live in urban (Heping, Hedong, Hexi, Nankai, Hebei, Hongqiao and Binhai New Area), suburban (Jinnan, Dongli, Xiqing and Beichen), or rural districts (Baodi, Wuqing, Ji, Jinghai and Ninghe). A finer administrative division in Tianjin is the urban community or rural village, with Tianjin residents registered to live in one of the 5,073 communities/villages which collectively comprise the entire municipality.

This case-control study compares cases sampled from the infectious disease surveillance system in Tianjin with a random sample of controls. Cases had laboratory confirmation (based on detection of IgM antibodies, a 4-fold increase in serial IgG titers, or isolation of viral RNA). Each week, staff at the Tianjin Centers for Disease Control and Prevention (CDC) downloaded the list of measles cases recorded within the past week from the China Information System for Disease Control and Prevention and randomly selected cases to enroll into this study based on overall measles case-load. For weeks with fewer cases, most cases would be enrolled, for weeks with high case counts (i.e., ≥40 cases), usually in the late winter early spring, only a fraction would be approached for enrollment. Between 1 and 37 cases were enrolled each week.

Our community controls were selected using a population-based, two-stage cluster design. At the first stage, 120 communities/villages were selected through a probability proportionate to size procedure with each district represented by at least one community/village. Between 12 and 32 people aged 1–49 years were enrolled from each community/village’s population registry using an age-stratified random selection procedure. Women aged 20–39 years who had infants were oversampled.

Population registries in Tianjin include both locals and non-locals (i.e. internal migrants to Tianjin) whose residency cards, or *hukou*, show that they are originally from another province. Insurance is tied to an individual’s home province of record in China, so non-locals have more difficulty accessing health care [12]. The *hukou* is passed down through family lines and can be difficult to switch.

Adult participants gave informed consent, and parents gave consent for their minor children, before any data collection occurred. Participants were interviewed in person. The interview included questions about socio-demographic characteristics, vaccination history, measles infection history, and exposure to various congregate settings in the prior 21 days (for controls) or 21 days prior to disease (for cases).

Participants were asked about hospitals they attended in the 21-day exposure period. Patients generally present first at township hospitals, and are sequentially transferred to higher-level facilities, i.e. district and then municipal hospitals. Municipal hospitals are the largest and tend to employ the most highly educated staff. Other specialty hospitals included infectious disease and pediatric hospitals.

Bloodspots were obtained by lancing the participant's finger with a single-use lancet, dropping the blood onto filter paper, and drying at room temperature. After being transported to the Tianjin CDC laboratory, dried bloodspots were tested for measles IgG antibodies [13]. IgG testing was done with a SERION ELISA measles virus IgG quantitative kit (Institut Virion/Serion GmbH, Würzberg, Germany). The IgG titers were categorized as positive if > 200 IU/ml, borderline if 150 to 200 IU/ml, and negative if <150 IU/ml [14].

Individuals who are measles IgG positive are assumed to be protected against future infection. For this reason, we include two control groups in our analysis: one comprising all individuals from the community sample (positive, borderline and negative), and the second consisting of only those who are IgG negative.

### Statistical analysis

Counts and weighted proportions were calculated to describe the distribution of risk factors in the cases, community controls, and IgG-negative controls. Survey weights were created through a raking procedure so that the cases were representative of all cases from the surveillance system in terms of sex, age category, urbanicity and residency; similarly, weights for the community controls were constructed to be representative of the general population based on Census 2010 figures for sex, age category, urbanicity and residency.

To create parsimonious multivariable logistic regression models predicting measles case status and to compare which variables would be entered into a model using either IgG-negative controls or all community controls, we utilized a best subsets selection technique with the branch-and-bound algorithm of Furnival and Wilson [15]. All independent variables were *a priori* thought to be possible risk factors for measles. We allowed 1 to 20 variables to be selected into the model. The same variable selection algorithm was used for both models (with IgG-negative controls and with all community controls): the final model included the variables in the largest model after which there was an increase of less than 3.84 in the likelihood score statistic (corresponding to *P* = 0.05 for one added variable). If any one indicator of a categorical variable was selected to be in the model, all other categories of that factor except the reference value were included. To demonstrate the consistency of choosing each variable in the model as more variables were added, we present the variable list for the ten models with the highest likelihood score statistics for 5 different model sizes: 2, 5, 10, 15, and 20 variables. The purpose of creating adjusted models this way is to compare (1) point estimates between the model with IgG-negative controls vs the model with all community controls, as well as (2) the specific variables that would be selected into each model. Significance was assessed at α = 0.05.

Odds ratios (ORs) are presented with 95% confidence intervals (CIs). OpenClinica version 3.3 (OpenClinica LLC, Waltham, MA, USA) was the database software used in the study. Data analyses were conducted using SAS software, version 9.3 (SAS Institute Inc., Cary, NC, USA).

### Ethics statement

This study was approved by the University of Michigan Institutional Review Board and the Tianjin Centers for Disease Control and Prevention Ethics Committee.

## Results

**Fig 1:**
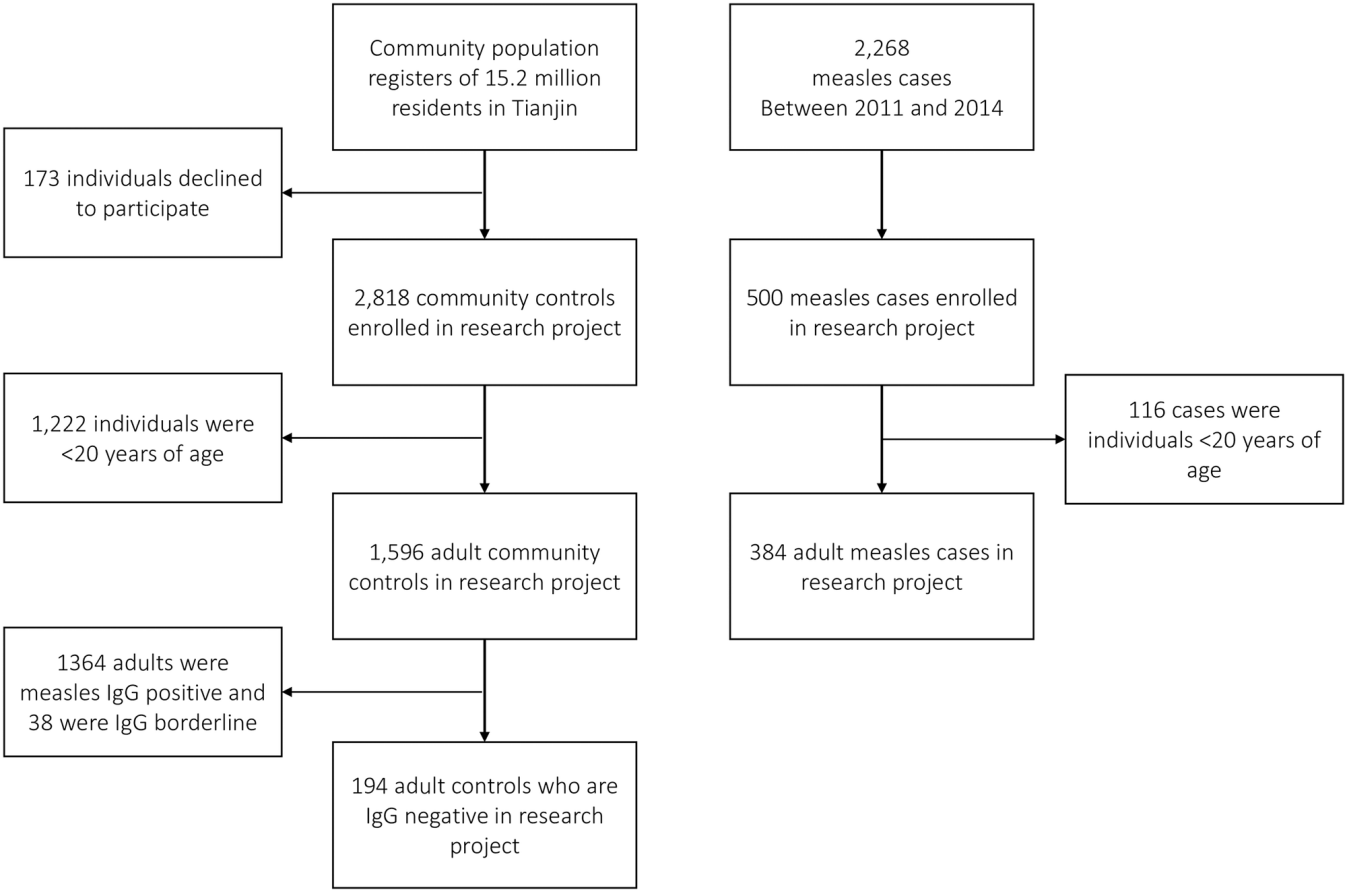
Diagram of how control and cases were enrolled into the study.

Fig 1 details the selection scheme. The study included 1,980 adults 20 to 49 years of age: 384 cases and 1,596 community controls, of whom 194 were measles IgG negative (Table 1). Approximately one-third (33.5%) of community controls had visited a hospital during the exposure period, compared to 49.1% of cases. A large majority of community controls (74.0%) did not know if they previously had measles or the measles vaccine. Only 15.3% of adult cases had a known contact with another measles case.

**Table 1:** Demographic characteristics among measles cases and two types of controls (IgG-negative controls and all controls), Tianjin, China, 2011–2015.

		Measles cases		IgG-negative controls		All controls	
		Count	Weighted %	Count	Weighted %	Count	Weighted %
**Overall**		384		194		1596	
**Age**	20–29 years	97	43.5%	92	38.0%	827	40.0%
	30–39 years	192	43.0%	92	43.6%	560	29.0%
	40–49 years	95	13.6%	10	18.4%	209	31.0%
**Sex**	Male	201	53.2%	39	63.8%	274	52.8%
	Female	183	46.8%	155	36.2%	1322	47.2%
**Urbanicity**	Urban	235	53.2%	105	58.3%	819	52.1%
	Suburban	85	21.6%	40	21.9%	279	19.9%
	Rural	64	25.2%	49	19.8%	498	28.1%
**Residency**	Local	299	78.4%	168	76.3%	1394	81.0%
	Non-local	85	21.6%	26	23.7%	202	19.0%
**Length of time in Tianjin**	≤3 years	43	12.6%	8	7.5%	81	5.7%
	>3 years	341	87.4%	186	92.5%	1515	94.3%
**Travel within China**	No	374	96.8%	180	89.2%	1488	90.3%
	Yes	10	3.2%	14	10.8%	108	9.7%
**Commute between home and work**	Within a community	240	65.2%	145	71.5%	1259	76.1%
	Between communities within a district	135	32.9%	21	13.6%	156	12.8%
	Between districts	9	1.9%	28	14.9%	181	11.1%
**Household size**	<4 people	275	69.9%	88	50.9%	731	51.6%
	≥4 people	109	30.1%	106	49.1%	865	48.4%
**Spent time on bus/train**	No	273	71.1%	133	64.4%	1087	67.4%
	Yes	111	28.9%	61	35.6%	509	32.6%
**Spent time at school/work**	No	177	47.3%	120	49.5%	943	51.2%
	Yes	207	52.7%	74	50.5%	653	48.8%
**Spent time in other congregate setting**	No	168	42.2%	81	35.7%	621	34.5%
	Yes	216	57.8%	113	64.3%	975	65.5%
**Occupation**	Professional	90	21.5%	56	35.0%	482	31.5%
	Service worker	39	10.2%	19	10.2%	155	10.0%
	Worker	154	41.5%	44	29.4%	407	30.9%
	Other	101	26.7%	75	25.3%	552	27.6%
**Spent time in hospital**	No	200	50.9%	124	65.1%	1030	66.5%
	Township hospital	27	7.2%	28	13.4%	228	12.1%
	County hospital	57	17.5%	11	5.5%	119	7.3%
	Municipal hospital	60	13.8%	20	10.6%	140	10.0%
	Specialty hospital	40	10.6%	11	5.3%	79	4.0%
**Known contact with measles case**	No	326	84.7%	192	97.8%	1582	99.2%
	Yes	58	15.3%	2	2.2%	14	0.8%
**Measles IgG status**	Positive	368	96.2%	0	0%	1364	86.0%
	Negative	15	3.6%	194	100.0%	194	11.8%
	Borderline	1	0.1%	0	0%	38	2.3%
**Immunizing history**	Disease and vaccination	18	5.4%	1	0.2%	25	1.1%
	Disease, not vaccination	366	94.6%	7	4.2%	79	5.8%
	Vaccination, not disease	0	0%	20	8.3%	217	14.9%
	Unknown	0	0%	135	74.0%	1036	61.7%
	Neither disease nor vaccination	0	0%	31	13.4%	239	16.5%

There were several differences observed when constructing regression models using IgG-negative controls vs. all controls. In the unadjusted analyses, 8 point estimates were statistically significant when using IgG-negative controls, compared to 14 when using all controls, although two of these point estimates related to vaccination, which was not assessed for models only using IgG-negative controls (Table 2). Table 3 shows the variables most often chosen into models according to a best subsets selection procedure. Results for model sizes of 5, 10, 15, and 20 variables are shown. For both sets of controls (IgG-negative controls and all community controls), commute between home and work, household size, occupation, and hospital visits were often selected to be in a multivariable model. Other variables were differentially selected into the two sets of models (Table 3).

**Table 2:** Risk factors for measles based on logistic regression using two different control groups: IgG-negative controls and all controls, Tianjin, China, 2011–2015. Survey weights were used.

		IgG-negative controls		All controls	
		Unadjusted model OR (95% CI)	Adjusted model OR (95% CI)	Unadjusted model OR (95% CI)	Adjusted model OR (95% CI)
**Age**	20–29 years	ref	ref	ref	ref
	30–39 years	0.86 (0.51, 1.45)	0.81 (0.44, 1.50)	**1.36 (1.01, 1.84)**	1.36 (0.95, 1.94)
	40–49 years	0.64 (0.30, 1.39)	0.45 (0.18, 1.14)	**0.40 (0.29, 0.57)**	**0.32 (0.20, 0.49)**
**Sex**	Male	ref		ref	
	Female	**1.61 (1.01, 2.56)**		0.90 (0.68, 1.19)	
**Urbanicity**	Urban	ref		ref	
	Suburban	1.13 (0.55, 2.34)		1.00 (0.69, 1.45)	
	Rural	1.67 (0.84, 3.32)		0.96 (0.67, 1.38)	
**Residency**	Local	ref		ref	
	Non-local	1.21 (0.58, 2.51)		1.28 (0.89, 1.82)	
**Length of time in Tianjin**	≤3 years	1.80 (0.60, 5.37)		**2.95 (1.79, 4.86)**	**2.08 (1.15, 3.75)**
	>3 years	ref		ref	ref
**Travel within China**	No	ref	ref	ref	ref
	Yes	**0.28 (0.10, 0.82)**	0.35 (0.10, 1.18)	**0.32 (0.14, 0.70)**	**0.25 (0.11, 0.58)**
**Commute between home and work**	Within a community	ref	ref	ref	ref
	Between communities within a district	**2.66 (1.31, 5.40)**	**3.30 (1.46, 7.45)**	**2.99 (2.15, 4.16)**	**3.37 (2.23, 5.10)**
	Between districts	**0.14 (0.06, 0.37)**	**0.20 (0.07, 0.63)**	**0.20 (0.10, 0.44)**	**0.24 (0.11, 0.54)**
**Household size**	<4 people	ref	ref	ref	ref
	≥4 people	**0.45 (0.26, 0.79)**	**0.35 (0.20, 0.60)**	**0.49 (0.36, 0.67)**	**0.33 (0.24, 0.46)**
**Spent time on bus/train**	No	ref		ref	
	Yes	0.68 (0.39, 1.18)		0.80 (0.59, 1.08)	
**Spent time at school/work**	No	ref		ref	
	Yes	1.08 (0.63, 1.85)		1.29 (0.96, 1.72)	
**Spent time in other congregate setting**	No	ref		ref	
	Yes	0.65 (0.37, 1.14)		**0.70 (0.52, 0.93)**	
**Occupation**	Professional	ref	ref	ref	ref
	Service worker	1.54 (0.61, 3.90)	1.98 (0.75, 5.26)	1.48 (0.90, 2.43)	**2.33 (1.33, 4.11)**
	Worker	**2.27 (1.17, 4.39)**	**3.40 (1.57, 7.40)**	**1.96 (1.39, 2.78)**	**3.15 (2.00, 4.94)**
	Other	1.62 (0.78, 3.35)	**2.52 (1.15, 5.53)**	1.15 (0.75, 1.76)	**2.01 (1.26, 3.20)**
**Spent time in hospital**	No	ref	ref	ref	ref
	Township hospital	0.55 (0.23, 1.31)	0.73 (0.33, 1.63)	0.64 (0.36, 1.14)	0.88 (0.48, 1.60)
	County hospital	**3.73 (1.14, 12.23)**	**5.19 (2.08, 12.92)**	**3.03 (1.90, 4.84)**	**4.13 (2.52, 6.76)**
	Municipal hospital	1.38 (0.59, 3.21)	**3.89 (1.49, 10.16)**	**1.65 (1.07, 2.54)**	**2.86 (1.73, 4.72)**
	Specialty hospital	**3.58 (1.26, 10.15)**	2.92 (0.93, 9.20)	**4.25 (2.43, 7.43)**	**3.93 (2.18, 7.08)**
**Measles vaccination**	Yes	—	—	**0.19 (0.10, 0.36)**	**0.14 (0.07, 0.27)**
	Unknown	—	—	**0.59 (0.42, 0.81)**	**0.43 (0.30, 0.61)**
	No	—	—	ref	ref

OR, odds ratio; CI, confidence interval

**Table 3:** Number of models that included each variable according to a best subsets selection, by model size. Results from the ten highest likelihood score statistics for each model size. For example, for models using IgG-negative controls and each limited to 5 explanatory variables, the variable ‘visited county hospital vs not’ appeared in 9 of the 10 models which had the highest likelihood score statistics.

		Cases–IgG-negative controls: Model size				Cases–all controls: Model size			
		5	10	15	20	5	10	15	20
**Age**	Age 30–39 years vs not	0	0	1	9	0	0	3	10
	Age 40–49 years vs not	1	5	10	10	10	10	10	10
**Sex**	Female vs male	2	6	10	10	0	0	1	5
**Urbanicity**	Suburban vs not	0	0	1	9	0	0	1	5
	Rural vs not	1	0	4	9	0	0	0	3
**Residency**	Non-local vs local	0	0	9	9	0	0	1	5
**Length of time in Tianjin**	Length in Tianjin ≤3 years vs not	1	2	10	10	1	3	10	10
**Travel within China**	Traveled within China vs did not	1	6	9	9	1	5	10	10
**Commute between home and work**	Home and work in same district vs not	10	10	10	10	10	10	10	10
	Home and work in different district vs not	10	10	10	10	1	4	8	10
**Household size**	≥4 people in household vs <4 people	10	10	10	10	9	10	10	10
**Spent time on a bus/train**	Spent time on bus/train vs not	0	0	1	9	0	0	2	10
**Spent time at work/school**	Spent time at work/school vs not	0	0	1	9	0	0	1	10
**Spent time in other congregate setting**	Spent time in other congregate setting vs not	0	0	1	9	0	1	2	9
**Occupation**	Other type of worker vs not	0	7	10	10	0	2	10	10
	Service worker vs not	0	3	9	9	0	0	10	10
	Worker vs not	1	10	10	10	2	9	10	10
**Spent time in hospital**	Visited county hospital vs not	9	10	10	10	4	10	10	10
	Visited municipal hospital vs not	1	10	10	10	0	6	10	10
	Visited specialty hospital vs not	1	10	10	10	2	10	10	10
	Visited township hospital vs not	2	1	4	9	0	0	1	3
**Measles vaccination**	Received measles vaccine vs not	—	—	—	—	8	10	10	10
	Unknown if received measles vaccine vs not	—	—	—	—	2	10	10	10

Table 2 shows results from multivariable logistic regressions modeling the relationship between various risk factors and measles cases status. In the model with cases and IgG-negative controls, living and working in separate communities within the same district of Tianjin was associated with 3.30 times higher odds of measles case status (95% CI:1.46, 7.45) and those living and working in separate districts had decreased odds of measles case status (OR = 0.20, 95% CI:0.07, 0.63), compared to those living and working in the same community. Larger households (≥4 people) had lower odds of measles case status (OR = 0.35, 95% CI:0.20, 0.60) compared to smaller households, and workers had greater odds of measles (OR = 3.40, 95% CI:1.57, 7.40) than those in a professional occupation. Visiting a hospital in the previous 21 days was a risk factor for disease; for example, visiting a municipal hospital was associated with 3.89 times higher odds of measles case status (95% CI:1.49, 10.16).

Models comparing cases to all controls had some similar findings, but in general had more precise confidence intervals. Using all controls, the comparison in odds of measles case status between households with ≥4 people vs <4 households was 0.33 (95% CI:0.24, 0.46); but using IgG-negative controls, the confidence interval increased (OR = 0.35, 95% CI:0.20, 0.60). Age (for adults 40–49 years compared to younger adults 20–29 years) was significantly related to measles case status when using all controls but not when using IgG-negative controls.

## Discussion

This study of measles cases and controls over a 5-year period in Tianjin, China, showed that travel patterns, household size, and exposure to hospital settings were all important predictors of measles case status, regardless of the control group used. Age and length of time in Tianjin demonstrated dissimilar relationships with measles case status depending on the control group used, which further underscores the importance of the selection criteria used for controls in a case-control study.

The strength of the association between exposure to a hospital and measles case status was strongest for specialty hospitals. This may reflect the extra time that an individual spends at one or more hospitals before admission to a specialty hospital. Xiong et al.’s 2006 study from Jilin province found the odds of measles to be 9.7 times higher for those with visits to 2 separate hospitals (95% CI:2.6, 38) but only 5.4 times higher with a visit to only one hospital (95% CI:1.9, 16) [16]. Measles cases are likely to be present at infectious disease hospitals in China, and unless strict infection control and isolation measures are in place, could serve a venue for transmission particularly if patients are placed in aggregate settings like waiting rooms. It is especially concerning that infectious disease hospitals were a significant risk factor for measles because ostensibly these hospitals should be highly attuned to the potential of disease transmission. The use of telemedicine has been increasing in China, particularly in more remote regions [17], and having infectious disease doctors consult with parents and patients via internet video could be one way to minimize contact among potentially infectious individuals.

Travel outside of the city was a preventive factor. Other studies in China have shown mixed results for the relationship between travel and measles [8,9]. Travel could be a marker of affluence, which itself could be negatively associated with measles case status: e.g., (blue collar) workers were at greater risk for measles than professional workers. Similarly, living and working in different districts was preventive, whereas living and working in different communities within the same district was a risk factor. This difference is also plausibly due to socioeconomic differences between people who travel farther vs less far for work: more affluent individuals may have the means to live the furthest away from their work, whereas the least affluent may travel a shorter distance for work, and may rely more on public transportation.

Household size may be causally related to measles acquisition through several mechanisms. Larger households plausibly have more opportunities for measles exposure because of the close contact of people in close quarters [18], but we observed the opposite trend. We did not collect information on household composition (i.e., number of children or grandparents within the household), but presumably families of ≥4 people represent families where at least one grandparent is in the household, given that the One Child Policy was in force during the study period. An extended family may be protective for a number of different reasons: the family may have more ties to Tianjin and less likely to have contact with people outside the municipality or these households may be socioeconomically different than nuclear families.

### Recommendations for selection of control participants

Previous case-control studies of measles in China have included controls without regard for antibody status [7]. We were unable to identify literature from any other country that compared controls from the general population versus those who are IgG negative. A model using IgG-negative controls will have a smaller sample size but will minimize bias. A model using all controls will have a larger sample size yielding greater power but could have substantial bias, depending on whether differences between IgG negative and IgG positive controls have a different distribution of risk factors related to measles. Some may misconstrue using all controls as the better option because of statistical power while neglecting the possibility of bias [19].

There were some differences between the two sets of models in our study. Adults 45–49 years of age had lower odds of measles (and adults 40–44 marginally so) in the model with all controls, but not the model with IgG-negative controls. This follows from a larger proportion of the older adults being IgG positive (probably due to timing of the introduction of the immunization program [4], and a higher probability of obtaining immunity through disease [5]); i.e., a model with all controls is biased towards older adults, who will tend to be less susceptible leading to observing a lower risk of measles, whereas in the model with IgG-negative controls, this effect is absent. In addition to differences in susceptibility among IgG-positives by age, other variables such as travel patterns, public transportation use, or different places for work and leisure may vary with age and further confound the intended effects [20].

The model with IgG-negative controls did not select residency or length of time in Tianjin as a risk factor for measles, but length of time in Tianjin (but not residency) was selected in the model with all community controls. Other literature from China has shown a relationship between residency and measles case status [7,9], even going as far to call non-locals, or the floating population, the “primary reservoirs” for measles transmission [21]. But non-locals represent a heterogeneous group in the context of employment and income, and there are differing characteristics among non-locals in regards to when they migrated and where they are originally from [22]. In the model comparing cases to IgG-negative controls, length of time in Tianjin was not associated with measles case status, and although this could result from a smaller sample size, it also may be indicative of the model with all controls showing spurious results by including shorter-term residents who are IgG positive. In essence, studies wishing to identify infectious disease patterns would be better served by using IgG-negative controls. Studies aiming to identify group for vaccination or other preventive control measures could use all controls regardless of immunity, but these findings could also be obtained through a seroprevalence study.

### Strengths and limitations

This study is cross sectional, and it is plausible that cases may recall their exposure history differently than controls, with controls perhaps more likely to underreport hospital visits. The high proportion of adults who had an unknown vaccination status is a limitation of any study looking to identify predictors of case status, however, by examining antibody results, we were able to ascertain who was in the source population of cases. Additionally, collider bias, which results from exposure and outcome causing the sampling scheme, easily occurs in case-control studies because by definition we sample based on the outcome status. A strength of this study is that we sampled from all districts within Tianjin over a number of years, and so are able to generalize our results beyond the happenstance of a specific outbreak.

## Conclusions

Hospital visits and travel patterns were two modifiable risk factors for measles; the former can be mitigated through improving infection control in hospitals, but more research is necessary to discover how distinct modes of travel and different travel patterns result in disease, particularly in populations that have different motivations for travel, such as work, leisure, and visiting relatives.

The choice of an appropriate control group is a trade-off between bias and variance, where the use of a smaller number of (e.g., IgG negative) controls may better represent the source population, but produce results with higher variance due to the smaller sample size. Where feasible, we recommend using IgG-negative controls. As adults become an increasingly large proportion of measles cases in China, identifying risk factors for their acquisition of measles may require validation of IgG-negative control status through the use of antibody testing (e.g., in conjunction with a seroprevalence study).
